# Intraoperative Extracorporeal Support during Lung Transplantation: Not Just for the High-Risk Patient

**DOI:** 10.3390/jcm13010192

**Published:** 2023-12-29

**Authors:** Daniel Laskey, Brian Housman, Gbalekan Dawodu, Scott Scheinin

**Affiliations:** Thoracic Surgery Department, Icahn School of Medicine at Mount Sinai, Mount Sinai Health System, One Gustave L. Levy Place, Box 1023, New York, NY 10029, USA; brian.housman@mountsinai.org (B.H.); gbalekan.dawodu@mountsinai.org (G.D.); scott.scheinin@mountsinai.org (S.S.)

**Keywords:** single-lung transplant, double-lung transplant, pulmonary hypertension, rejection, complications, extracorporeal membranous oxygenation, ischemia-reperfusion injury

## Abstract

The use of intraoperative mechanical support during lung transplantation has traditionally been a controversial topic. Trends for intraoperative mechanical support strategies swing like a pendulum. Historically, cardiopulmonary bypass (CPB) was the modality of choice during transplantation. It provides full hemodynamic support including oxygenation and decarboxylation. Surgical exposure is improved by permitting the drainage of the heart and provides more permissive retraction. CPBs contain drainage reservoirs with hand-held pump suction catheters promoting blood conservation through collection and re-circulation. But CPB has its disadvantages. It is known to cause systemic inflammation and coagulopathy. CPB requires high doses of heparinization, which increases bleeding risks. As transplantation progressed, off-pump transplantation began to trend as a preferable option. ECMO, however, has many of the benefits of CPB with less of the risk. Outcomes were improved with ECMO compared to CPB. CPB has a higher blood transfusion requirement, a higher need for post-operative ECMO support, a higher re-intubation rate, high rates of kidney injury and need for hemodialysis, longer ICU stays, higher incidences of PGD grade 3, as well as overall in-hospital mortality when compared with ECMO use. The focus now shifts to using intraoperative mechanical support to protect the graft, helping to reduce ischemia-reperfusion injury and allowing for lung protective ventilator settings. Studies show that the routine use of ECMO during transplantation decreases the rate of primary graft dysfunction and many adverse outcomes including ventilator time, need for tracheostomy, renal failure, post-operative ECMO requirements, and others. As intraoperative planned ECMO is considered a safe and effective approach, with improved survival and better overall outcomes compared to both unplanned ECMO implementation and off-pump transplantation, its routine use should be taken into consideration as standard protocol.

## 1. Introduction

The use of intraoperative mechanical support during lung transplantation varies across transplant centers and has traditionally been a controversial topic. There is a lack of general consensus as strategies vary widely, partly due to an absence of substantial clinical trials, with discussion based upon retrospective reports with varied results. Historically, studies were unbalanced, comparing sicker patients in more severe conditions requiring extracorporeal life support to uncomplicated patient cohorts transplanted off-pump as the control group. The first serious experimentation for lung transplantation started in the beginning of the 20th century with Dr. Alexis Carrel, implanting heart–lung en bloc into the neck of cats [1]. These animal models continued on through mid-century, with meager, yet improving results. On 11 June 1963, the first lung transplant was performed on a human patient by Dr. James Hardy. This was a left lung transplanted into a patient with bronchial carcinoma who could not tolerate a pneumonectomy due to advanced emphysema [1]. The patient survived for 18 days before succumbing to renal failure and infection; however, on autopsy, the lungs were well ventilated without signs of rejection. The first heart–lung transplant was performed by Dr. Denton Cooley in 1968 in a 2-month-old infant. Subsequent heart–lung transplants were performed by Lillehei and Barnard, respectively. Outcomes continued to be marginal at best due to the lack of effectual immunosuppression. This continued on through the 1970s with 38 lung, lobe, or heart–lung transplants performed between 1963 and 1978 without significant long-lasting success [1].

Dr. Joel Cooper, from Toronto, in 1968, used a membrane oxygenator to support the transplantation of a right lung. The patient was a victim of a house fire and had persistently high pCO_2_ despite maximal ventilator support due to respiratory burns. The membrane oxygenator maintained the patient throughout the perioperative period. The patient survived until the third post-operative week and was able to be liberated from ventilator support, ultimately expiring from bronchial anastomosis dehiscence [1]. As immunosuppression improved, so did outcomes. Dr. Cooper et al. in Toronto successfully transplanted a right lung into a 58-year-old patient with idiopathic pulmonary fibrosis who survived for seven years.

Trends for intraoperative mechanical support strategies swing like a pendulum. Lung transplants were, and continue to be, performed primarily by cardiac-trained surgeons who are well accustomed to cardiopulmonary bypass. As a result, CPB was traditionally used routinely for intraoperative support, maintaining hemodynamic stability in high-risk patients [2]. However, as lung transplant continued to progress, the complications of cardiopulmonary bypass became more apparent, resulting in a trend towards off-pump transplantation. Recently, however, there has been a swing back towards routine intraoperative support in the form of veno-arterial ECMO with good outcomes.

## 2. Methods

A PubMed search for English-language articles exploring the use of intraoperative mechanical support in the setting of single- and double-lung transplantation was conducted. Key words included “mechanical support”, “single lung transplant”, “double lung transplant”, “extracorporeal mechanical support”, “cardiopulmonary bypass”, “complications”, “extracorporeal membranous oxygenation”, “death”, and all appropriate Boolean operators. While referencing historically significant studies for perspective, we prioritized recent research from prospective and retrospective studies that evaluated clinical outcome. Studies that discussed risk factors, mechanisms, and outcomes were also included, as were retrospective database research and other review articles. The guidelines, original works, and foundational studies from professional societies and individual leaders in the field were also reviewed. Only articles agreed upon by all authors were included.

## 3. Cardiopulmonary Bypass as Support

Cardiopulmonary bypass was the standard support in the early days of lung transplantation. It was described in the literature for use during lung transplantation as early as the 1990s. In 1994, Triantafillou et al. reviewed 162 lung transplants from 158 patients: 8 en bloc double-lung transplants, 83 single lung transplants, and 71 bilateral sequential lung transplants. Bypass was used electively for all double en bloc transplants, 3 of the bilateral sequential transplants, and for 26 unilateral transplants [3]. In 1993, de Hoyos et al. described 69 patients, 38 single-lung transplants and 31 double-lung transplants. Of these patients, 12 of the single-lung transplants and 10 of the double-lung transplants required cardiopulmonary bypass [4].

Cardiopulmonary bypass provides full hemodynamic support including oxygenation and decarboxylation [5], allowing a high-risk patient, with pulmonary hypertension, severe hypoxia, or poor ventilator status, to undergo an otherwise perilous procedure, clamping the pulmonary artery, and pneumonectomy, in a stable fashion. Surgical exposure is improved by permitting the drainage of the heart and provides more permissive retraction. CPBs contain drainage reservoirs with hand-held pump suction catheters promoting blood conservation through collection and re-circulation. In addition, with CPB, concomitant intra-cardiac surgeries at the time of transplantation can be performed, opening the recipient pool to more complex and comorbid patients. Lastly, CPB allows the controlled reperfusion of the transplanted lung, a major factor in preventing primary graft dysfunction.

## 4. Disadvantages of Cardiopulmonary Bypass

However, CPB also has disadvantages. It is known to cause systemic inflammation and coagulopathy through the interaction of the patient’s blood with the non-endothelial surface of the pump circuit, the coagulation cascade, and the complement system [6]. A coagulation cascade is activated through an immunomodulatory effect leading to pro-inflammatory cytokine release such as tumor necrosis factor alpha and interleukin 6 [7,8,9,10]. Some patients are found have an exaggerated inflammatory response to CPB, leading to increased bleeding, increased ventilator requirements, increased capillary leak and associated edema, and an overall decline of independent functioning [9]. Coagulopathy induced by CPB is largely associated with the non-laminar flow in the oxygenator and blood stasis in the venous reservoir [11]. The consumption of coagulation factors and von Willebrand factor [10] with associated platelet dysfunction is common. Hemodilution from the priming fluid can also cause a decrease in platelets contributing to the coagulopathy. Endothelial activation leads to tissue plasminogen activator causing fibrinolysis [11,12,13].

Cardiopulmonary bypass requires high doses of heparinization, which increases bleeding risks [14,15]. Protamine, the reversal agent for heparin given at the end of the bypass, is linked to several adverse reactions including a paradoxical anticoagulation effect at excessive doses, anaphylaxis, pulmonary edema, and pulmonary hypertension [15]. Heparin rebound can also occur after protamine reversal, leading to an increased bleeding risk. Prolonged time on CPB is associated with worse overall outcomes, including mortality [16]. Also specifically with lung transplantation, there is a concern that the suctioning of the contaminated open bronchial system into the reservoir system during CPB may seed bacteria into the circulation, leading to bacteremia in an immunosuppressed patient.

## 5. Cardiopulmonary Bypass versus Off-Pump

As transplants became more successful, with improved techniques, post-operative management, and better outcomes reported, the trend transitioned to performing transplants off-pump, with research supporting this; there was no strong evidence that CPB was necessary for a successful transplant. Dog models using CPB in single-lung transplantation had poor outcomes: impaired gas exchange, hemodynamic instability, and increased blood loss [17,18]. Data from human transplantation were less consistent. In 2010, Negendran et al. reviewed 14 papers asking whether double-lung transplantation should be performed with or without cardiopulmonary bypass. There was very little Level 1 evidence. All 14 papers were retrospective. Six of the papers found significantly worse outcomes with CPB use, six found no difference, and two found a mixture of both [19].

In 2016, Mohite et al. compared the outcomes of 302 lung transplants by dividing the patients into three groups: 86 patients in the off-pump group, 162 patients in the elective-CPB group, and 54 patients in an unplanned conversion to CPB group. The off-pump group had significantly better arterial oxygen partial pressure (PaO_2_) to fractional inspired oxygen (FiO_2_) ratio (P/F ratio). In addition this group had a significantly shorter duration of mechanical ventilation, ICU stay, and hospital length of stay compared to propensity-matched elective-CPB patients [20]. The unplanned emergent group had worse cumulative survival in one, two, and three years compared to both the elective and the off-pump cohorts [20].

As developments in transplantation continued to increase, so did the complexity of patients [21]. Higher allocation scores, older patients, patients with more comorbidities, patients with complex pulmonary fibrosis, pulmonary hypertension, re-transplantation, and multi-organ transplantation were being included in the recipient pool [22]. These more aggressive donor and recipient selection strategies resulted in higher instances of primary graft dysfunction [23].

## 6. ECMO versus Cardiopulmonary Bypass

Is VA-ECMO a better solution? ECMO provides a miniaturized closed circuit compared to CPB without the air-blood interaction found in a CPB reservoir. Given this smaller circuit, there is less inflammatory activation with minimal coagulative disorders. There is a lower primer volume compared to CPB, reducing hemodilution. ECMO also has a smaller heparin requirement, which decreases the bleeding risk [24].

In 2012, Ius et al. [25] compared patients transplanted with intraoperative CPB compared to VA-ECMO: 46 patients in the CPB group versus 46 patients on VA-ECMO. The ECMO group had a more severe risk profile, including patients with pulmonary hypertension, pre-operative intensive care stays, and bridging with extracorporeal life support (ECLS). Survival, the failure of secondary organs, bleeding complications, and the need for blood/platelet transfusions were evaluated. The cardiopulmonary bypass group had higher in-hospital mortality, need for hemodialysis, and new post-operative ECMO support compared to the ECMO group, as well as an increased number of packed RBC and platelet transfusions. A multivariate analysis identified CPB as an independent risk factor for in-hospital deaths. Long-term survival was significantly impaired in the CPB group while the ECMO group was comparable to off-pump transplantation.

In 2014, Bermudez et al. [26] performed a retrospective study of 271 patients undergoing lung transplantation. A total of 222 transplantations were carried out using CPB and 49 with VA-ECMO. Of the CPB group, 24.5% received concurrent cardiac procedure. Both groups had comparable demographics and operative characteristics. The ECMO group had a high lung allocation score (LAS) as well as a longer average time on support and a higher percentage of patients bridged with ECLS and lobar transplants, but did not include patients with pulmonary artery hypertension. Despite this, the cardiopulmonary bypass group had a higher re-intubation rate and number of temporary tracheostomy, as well as an increased frequency of renal failure requiring hemodialysis. There was no difference in rates of severe primary graft dysfunction (PGD) necessitating post-operative circulatory support or the need for perioperative blood transfusion. In addition, there was no difference in 30-day or 6-month mortality between the groups.

Biscotti et al. [27] also performed a retrospective analysis in 2014, comparing 55 patients transplanted on CPB versus 47 on VA-ECMO. The cardiopulmonary bypass group had higher rates of PGD at the 24 and 72 h time points, and they were more likely to have bleeding and require re-operation compared to the ECMO group. The one-year survival was the same in both groups.

Machuca et al. [28] performed a further comparison in 2015 between 66 CPB cases and 33 ECMO cases. The patients in both groups had similar variables: age, BMI, last PaO_2_/FiO_2_ ratio, smoking history, positive airway cultures, and donor type (i.e., brain death and donor after cardiac death). Early outcomes were reviewed, with mechanical ventilation requirements, length of ICU stay, and length of hospital stay all significantly favoring ECMO. There were significantly more perioperative blood product transfusion requirements in the cardiopulmonary bypass group and a higher rate of revisions due to bleeding complications. A non-significant trend for higher rates of dialysis requirements, the need for post-operative ECMO, and 90-day mortality was observed in the cardiopulmonary bypass group.

In 2015, the Munich Lung Transplantation Group [29] reviewed 49 patients with extracorporeal circulatory support during transplant: 22 on cardiopulmonary bypass and 27 on VA-ECMO. The patients had comparable allocation scores and the principle indication for intraoperative support was the same in both groups: pulmonary hypertension. The cardiopulmonary bypass group had a significantly higher intraoperative transfusion requirement compared to the ECMO group: 9 units pRBC (5–18) in the CPB group compared to 6 units (4–8) in the ECMO group. Other blood products were similar: 3.5 units platelets (2–4) versus 2 units (0–3), 5 g fibrinogen (4–6) versus 0 g (0–4), 3 iU prothrombin complex concentrate (2–5) versus 0 iU (0–2), and 92.5 mg tranexamic acid (2–5) compared to 2.0 mg (1.3), respectively. Ventilator support requirements were longer in the cardiopulmonary bypass group: 21 days (7–31) versus 5 days (3–21). The length of intensive care stays were markedly longer in the cardiopulmonary bypass group at 36 days (14–62) compared to 15 days (6–44) in the ECMO group. There was, however, no difference in 30-day and 1-year mortality rate.

In 2020, Dell’Amore’s [30] group evaluated extracorporeal life support use during bilateral sequential lung transplantation in patients with pulmonary hypertension. A total of 17 cases were performed with CPB and 21 cases with ECMO. Of these patients, 8 of the CPB and 7 of the central ECMO patients, i.e., 15 patients in total, continued peripheral ECMO post-operatively to the ICU. The researchers found that intraoperative blood requirements were higher in the cardiopulmonary bypass group, with a median of 4 units packed red blood cells, compared to 2 units in the ECMO group. In addition, the transfusion requirement in the first 72 h post transplant was decreased in the ECMO group compared to the CPB group: a median of 4 units pRBC in the CPB group and 1 unit pRBC in the ECMO group. In the group requiring prolonged post-operative ECMO in the ICU, a median of 5 units pRBC were required if the patient had intraoperative cardiopulmonary bypass compared to 2 units pRBC if intraoperative central ECMO was utilized. The mean duration of post-op prolonged ECMO was not significantly different between the CPB and the intraoperative ECMO group; however, the median ventilation time was higher in the patients supported by cardiopulmonary bypass. In addition, the ICU length of stay was longer in the CPB group and shorter in the ECMO group. Lastly, the incident of acute renal failure as well as primary graft dysfunction (PGD) grade 3 at 72 h were both lower in the ECMO group compared to the CPB group, and in-hospital mortality was also lower.

Recently, in 2022, the Extracorporeal Life Support in Lung Transplantation Registry conducted an analysis of a multicenter international registry looking at double-lung transplants performed at eight high-volume centers [31]. The association between modes of extracorporeal support and the incidence of PGD grade 3 was compared. A total of 852 transplants were reviewed between January 2016 and March 2020. Of these, 422 transplants (50%) were performed off-pump, 273 (32%) with ECMO, and 157 (18%) with CPB. The PGD rates at time point 48–72 h were 12.1% for off-pump, 28.9% for ECMO, and 42.7% for CPB. ECMO was associated with PGD grade 3 more than off-pump was, but less than cardiopulmonary bypass was, indicating that if extracorporeal life support is required, then ECMO is preferred.

To summarize, CPB has a higher blood transfusion requirement, a higher need for post-operative ECMO support, a higher re-intubation rate, high rates of kidney injury and need for hemodialysis, longer ICU stays, higher incidences of PGD grade 3 [32], as well as overall in-hospital mortality when compared with ECMO use. The pendulum is shifting towards intraoperative ECMO to support the high-risk patients [33]. But what about the routine use of ECMO for all transplantations?

## 7. Routine Use of ECMO

Initial studies showed that outcomes were the same, if not better, when ECMO was used compared to off-pump transplantation. In 2016, Ius et al. [34] conducted a 5-year retrospective study comparing 425 patients transplanted off-pump versus 95 patients with elective ECMO, and 75 patients who required unplanned intraoperative ECMO. The study demonstrated no difference in long-term outcomes between off-pump and perioperative ECMO during transplants. The overall patient and graft survival was similar among patients who underwent transplantation with and without ECMO support. The post-operative complications and in-hospital mortality were high in the ECMO group in this study; however, these patients had significantly worse pre-operative conditions, with risk factors including high allocation scores, a higher percentage of pulmonary hypertension, and higher pre-operative mechanical ventilation, pre-operative ICU care, and pre-operative ECMO use.

## 8. Intraoperative Mechanical Support’s Role in Reperfusion Injury

Historically, intraoperative support was used primarily to provide complete control of hemodynamics and ventilatory status, especially in high-risk patients [35,36]. However, there has been a recent move towards creating a protective environment for the graft [37], especially during early reperfusion. Ischemia-reperfusion injury results from endothelial and epithelial injury releasing cytokines and triggering innate immune response, leading to a rapid inflammatory effect [38]. This clinically manifests as early graft dysfunction and non-cardiogenic pulmonary edema [36,39]. The injury and disruption to the endothelial barrier and subsequent inflammation are mediated by oxidative injury from reactive oxygen species created as a response to ischemia [38]. The slow release of the pulmonary artery during reperfusion has been demonstrated to be beneficial in reducing this injury. Animal models from the 1990s demonstrated that low-pressure reperfusion, at 50% physiological pressure, over 10 min prevented many of the injuries that were seen when reperfusion occurred at normal physiological pressure [39]. This was regardless of the preservation medium and the duration of the ischemia. Further animal models confirmed similar findings, with a reduction of ischemia-reperfusion injury following controlling initial perfusion pressure [40]. A total of 30 min of controlled reperfusion is superior to 15 or 5 min [41]. This was taken further, and controlled reperfusion was performed with incremental increases of pressure every 15 min for an hour. The rat models in this group achieved lung function similar to baseline values, compared to models which had significant dysfunction after immediate reperfusion at physiological pressures [42].

A continued delay with the use of intraoperative support can further diminish injury secondary to reperfusion by controlling the flow rate. ECMO provides stable intraoperative conditions and allows for controlled reperfusion. It can protect the first implanted lung from over-perfusion as the second lung is being transplanted, preventing first lung syndrome.

First lung syndrome occurs when the first implanted lung bears the entire cardiac output during the clamping of the contralateral pulmonary artery. This increases the reperfusion pressure, leading to graft dysfunction. Intraoperative mechanical support bypasses a significant portion of the pulmonary circulation, reducing this problem. ECMO protects the transplanted lung in the crucial phase when it is recovering from injury of procurement and ischemia. In addition, ECMO voids aggressive ventilation by allowing for a lung protective ventilation strategy with a low pressure and low tidal volumes.

Routine intraoperative ECMO use can avoid high FiO2 and high ventilation pressures during transplantation. Both are known risk factors for the ventilator-induced lung injury of the allograft and the subsequent development of PGD [32].

## 9. Intraoperative ECMO’s Role in the Modern Era

The routine use of ECMO avoids the need for acute, unplanned ECMO insertion during transplantation. In a study of 436 patients who underwent double-lung transplantation, patients who had unplanned intraoperative ECMO were compared to patients without ECMO use and to those with planned usage. The one-year and three-year survival were lower in the unplanned ECMO group than in the no-ECMO group [43].

In 2018, Hoetzenecker et al. compared 118 patients transplanted without ECMO to 343 patients with elective intraoperative ECMO and 123 patients who underwent prolonged ECMO into the early post-operative period [44]. In the ECMO group, 1-year, 3-year, and 5-year survival were found to be superior compared to those of patients transplanted without ECMO use. A difference was noted in patient characteristics between the non-ECMO group and the intraoperative ECMO group, and therefore a propensity score matching was performed. Even with this matching, the 1-year, 3-year, and 5-year survival analysis was consistent. In addition, a trend towards a higher ratio of oxygen tension/fraction of inspired oxygen at ICU admission was found in the matched intraoperative ECMO group, and this was persistent post-operatively on day 1 after transplantation.

Despite the higher number of complex patients in the group who required prolonged ECMO post-operatively, the outcomes were excellent, with higher survival rates compared to the non-ECMO group at all time points. Prophylactic post-operative ECMO prolongation is associated with excellent outcomes in recipients with pulmonary hypertension and in patients with questionable graft function at the end of the implantation [44].

This same group, a few years later in 2020, investigated the routine elective use of ECMO during transplantation in a prospective observational study [45]. A total of 159 patients underwent bilateral transplantation on intraoperative ECMO with a PGD evaluated at 24, 48, and 72 h after transplantation. The grade 3 PGD rate at 72 h was extremely low at 1.3%. In addition, the 90-day mortality was very low at 3.1% and the 2-year survival was 86%.

Further investigation from Duke in 2022 [46] compared patients with no or mild pulmonary hypertension who underwent bilateral lung transplant either as planned off-pump, or with VA-ECMO support. A total of 237 transplants were included, 68 transplants using planned VA-ECMO and 169 transplants performed off-pump. These patients were evaluated for “textbook outcomes,” which was defined with the following criteria: freedom from intraoperative complications, 30-day re-interventions, grade 3 PGD at 48 or 72 h, post-transplant ECMO requirement, tracheostomy within 7 days, in-patient dialysis, reintubation, and extubation greater than 48 h post-transplant. A total of 20.6% (14 patients) of the planned VA EMCO group and 16.0% (27 patients) of the planned off-pump patients achieved textbook outcomes. Adjustments were made to account for prior lung transplantation, allocation score, ischemia time, and intraoperative transfusions. Despite this, the planned VA ECMO group had higher odds of textbook outcomes than the planned off-pump patients (odds ratio 3.89, 95% confidence interval 1.58–9.90).

## 10. Cannulation Strategies

Cannulation strategy is an important aspect of intraoperative extracorporeal life support [47]. There are two general strategies: central and peripheral cannulation. In a study by Glorion et al., evaluating outcomes associated with cannulation strategies in bilateral lung transplantation for pulmonary hypertension, the type of ECMO cannulation does not impact the in-hospital mortality or the long term survival rates [48]. Intraoperatively, during double-lung transplantation, central canulation is typically preferred as the mediastinal exposure allows for ease of access. Central cannulation provides adequate flows, a larger cannula to allow for optimal venous return, and room for intraoperative emergencies [48]. A single bolus dose of heparin, 40–60 U/kg, is required. The cannulation of the ascending aorta requires an 18 or 20 French single-stage cannula. Venous drainage is performed through atrial cannulation, and often a single-stage cannula is adequate; however, a double- or triple-staged cannula can be used. Central cannulation is typically placed with the expectation that it will be weaned off at the end of the procedure; however, prolonged central VA-ECMO cannulation can be secured tightly through skin incisions [49]. Generally, if prolonged ECMO is required, cannulation is converted to peripheral access.

Peripheral access is important in the case of sudden deterioration during induction and during single-lung transplantation when mediastinal exposure is limited [50]. Cannulation can be performed percutaneously or via surgical cut-down. Access tends to be through the femoral vessels [49]. The femoral artery is cannulated with a 15–19 French single-stage cannula. A 7 French distal perfusion cannula can be applied to avoid ischemia of the ipsilateral limb. The femoral vein is cannulated either ipsilaterally or contralaterally with a 21–25 French multi-stage cannula.

## 11. ECMO Complications

Complications during intraoperative extracorporeal life support can be significant and devastating to an already high-risk patient. The type and severity of the complication often depends on the cannulation strategies and the urgency of the initiation. Cannulations performed electively have fewer complications than those placed during an emergency. To avoid complications, a thorough assessment of the patient is conducted, including vascular imaging to evaluate for calcification, anatomic variants, and the size of the target vessels. Central cannulation is generally associated with fewer complications than peripheral cannulation, but this is speculated to be because central cannulation is generally discontinued at the end of the surgery, whereas peripheral cannulas may remain for a prolonged period post-operatively, leading to increased risk [48]. The complications of central cannulation include mediastinal bleeding, hematomas, and aortic dissections [48]. Care must be taken to avoid air embolism into the aorta. Peripheral cannulation complications tend to be related to target vessel size. The likelihood of dissection and hematomas is greater in smaller sized vessels [48]. There is a danger of groin infection, if the cannulation site remains post-operatively, and deep vein thrombosis, resulting in pulmonary embolism. Harlequin syndrome, due to cardiac output with poorly oxygenated blood competing with the arterial ECMO flows, resulting in diminished oxygenation to the upper portion of the body, is a risk of peripheral cannulation. In addition, distal ischemia of the ipsilateral limb is a significant complication of peripheral cannulation [51].

## 12. Conclusions

Throughout the history of lung transplantation, intraoperative mechanical support strategies ebb and flow with the tide of surgical techniques and advancements in the field. Initially performed entirely with the use of cardiopulmonary bypass machines, the trend switched to relying on mechanical support only for high-risk, complicated cases. Now, recent data have demonstrated that planned ECMO provides a successful graft-protective strategy and better outcomes, not only compared to unplanned ECMO use or in the setting of high-risk patients requiring prolonged post-operative ECMO requirements, but also in routine uncomplicated cases. As intraoperative planned ECMO is considered a safe and effective approach, with improved survival and better overall outcomes compared to both unplanned ECMO implementation and off-pump transplantation, its routine use should be taken into consideration as standard protocol.

## Data Availability

No new data was created—review article.
